# Microbial Primer: The R-pyocins of Pseudomonas aeruginosa

**DOI:** 10.1099/mic.0.001640

**Published:** 2025-12-10

**Authors:** Isaac Estrada, Parker Smith, Madeline Mei, Joanna B. Goldberg, Stephen P. Diggle

**Affiliations:** 1 Center for Microbial Dynamics & Infection, School of Biological Sciences, Georgia Institute of Technology, Atlanta, GA, USA; 2 Division of Pulmonary, Asthma, Cystic Fibrosis and Sleep, Department of Pediatrics, Emory University School of Medicine, Atlanta, GA, USA

**Keywords:** bacteriocins, phage tail-like bacteriocins, *Pseudomonas aeruginosa*, R-pyocins, tailocins

## Abstract

R-pyocins are phage tail-like protein complexes produced by *Pseudomonas aeruginosa* that deliver a single, lethal hit by depolarizing the target cell membrane. Unlike phages, R-pyocins lack capsids and DNA, and their killing is highly specific, being determined by tail fibre proteins that recognize subtype-specific LPS receptors on susceptible strains. Five known subtypes (R1–R5) vary in host range, with R5 displaying the broadest activity. R-pyocin expression is tightly regulated by the SOS response, linking their release to environmental stress. Their non-replicative mechanism and metabolic independence make them especially promising for targeting multidrug-resistant and biofilm-associated *P. aeruginosa* infections, such as those seen in cystic fibrosis and chronic wounds. Preclinical studies support their therapeutic potential, and bioengineering approaches have extended their target range. With their high specificity, rapid action and adaptability, R-pyocins are strong candidates for next-generation precision antimicrobials.

## Introduction

Pyocins are narrow-spectrum, proteinaceous antibiotics, or bacteriocins, synthesized by *Pseudomonas aeruginosa*. They were first described in 1945 as ‘pyocyanine-derived antibiotics’ and subsequently termed ‘pyocines’. These molecules confer competitive advantages by selectively targeting and eliminating closely related *P. aeruginosa* strains. Pyocins are categorized into distinct classes (S, L, F and R), each characterized by unique structural features and mechanisms of action. Among these, R-pyocins have garnered particular interest due to their potent, strain-specific bactericidal properties and structural resemblance to bacteriophage tails. Given their specificity and efficacy, R-pyocins represent promising therapeutic candidates against antibiotic-resistant *P. aeruginosa* strains, particularly in clinical contexts such as cystic fibrosis (CF) lung infections and other multidrug-resistant infections. This primer (1) outlines the structural and functional characteristics of R-pyocins (2); discusses the genetic regulation of their synthesis (3); delineates subtype classification and (4) summarizes recent developments regarding their therapeutic potential.

## Classification of pyocins and subtype diversity of R-pyocins

**Fig. 1.**
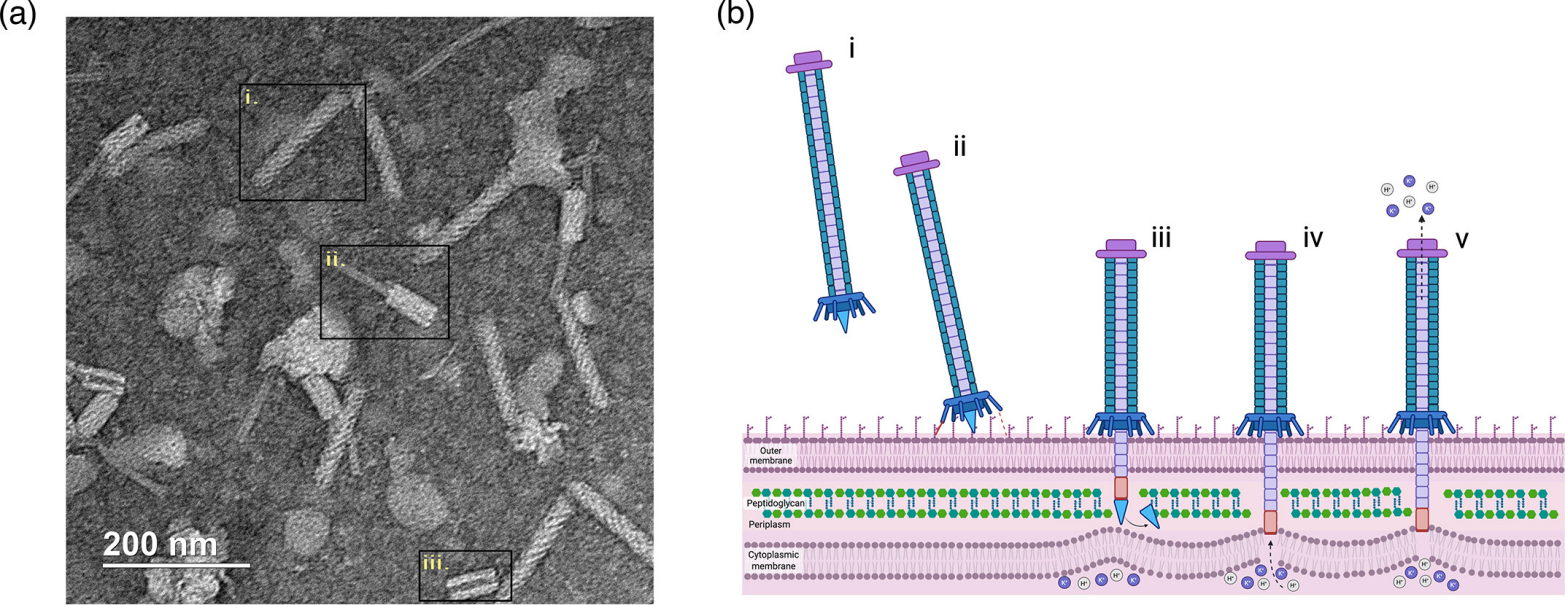
TEM visualization and schematic model of R-pyocin structure and killing mechanism in *P. aeruginosa*. (**a**) TEM image of purified R-pyocins. (**i**) Intact R-pyocin with an un-contracted sheath, capable of attaching to and killing a *P. aeruginosa* cell. (ii) Contracted R-pyocin with a protruding inner core tube, indicative of a post-firing state that can no longer bind to a target cell. (iii) Empty contracted sheath following the dissociation of the inner tube after firing. (**b**) Schematic model of the R-pyocin killing mechanism in *P. aeruginosa*. (**i**) When free in solution, the extended tail fibres of the R-pyocin (purple baseplate, long blue sheath and iron-tipped core) search for specific receptors on the bacterial outer membrane. (ii) Upon encountering its receptor, the tail fibres bind to defined monosaccharide residues (such as l-rhamnose or d-glucose) within the LPS outer core, stabilizing the complex on the cell surface. Binding triggers a rapid conformational change in the baseplate from a hexagonal to a star-shaped configuration, which initiates contraction of the tail sheath. (iii) The contracted sheath drives the rigid inner tube through the outer membrane, aided by enzymatic degradation of the peptidoglycan layer via the lysozyme domain of the tail spike. (iv) The iron-tipped core penetrates into close proximity to the cytoplasmic membrane, forming a transmembrane ion channel. (**v**) Dissipation of cations in the cytoplasm is aided by the negative charge of the R-pyocin tube, depolarizing the membrane by dissipating the proton-motive force. Loss of membrane potential rapidly collapses cellular energetics, leading to cell death without the need for nucleic acid injection. TEM, transmission electron microscopy.

*P. aeruginosa* strains typically encode multiple pyocin classes but usually express a limited subset. S-pyocins are soluble, single-protein toxins that frequently exhibit nuclease activity and are associated with specific immunity proteins to prevent self-toxicity. l-pyocins exhibit lectin-like properties, being structured as dimers comprising tandem mannose-binding domains. Contrastingly, F- and R-pyocins are structurally complex assemblies resembling phage tail-like appendages but lacking phage head components such as capsids and genomic material. F-type pyocins have a flexible, filamentous, non-contractile tail structure analogous to siphophage tails. In contrast, R-type pyocins are rigid, contractile structures that share a strong structural resemblance to *Myoviridae* phage tails, consisting of a sheath, core tube, baseplate and tail fibres (Fig. 1).

Each R-pyocin is further classified into distinct subtypes (R1–R5) based on variations in tail fibre protein sequences responsible for receptor specificity. These differences result in differential binding to distinct LPS core oligosaccharide residues on the targeted recipient bacterial cells. For instance, R1-pyocins specifically bind to l-rhamnose residues within the LPS core, whereas R2- and R5-pyocins interact selectively with d-glucose residues. Phylogenetic analyses divide R-pyocins into two major branches: R1 and R5 subtypes form one lineage, while R2, R3 and R4 subtypes cluster into another lineage with distinct receptor specificities.

Importantly, a single *P. aeruginosa* isolate typically harbours only one R-pyocin subtype gene cluster, and many isolates, including non-clinical strains, entirely lack R-pyocin genes yet often remain susceptible to R-pyocins. Among clinical isolates from respiratory infections, notably CF-associated isolates, the R1-type pyocin genotype often predominates. The spectrum of antibacterial activity varies considerably between subtypes, with R5-type pyocins displaying the broadest killing spectrum, R2-type pyocins having a narrower activity range and R1-type pyocins exhibiting the most restricted specificity. Despite this diversity, all R-pyocin subtypes are thought to share a conserved structural framework and mode of action, which involves recognition and binding to specific LPS receptors, followed by sheath contraction and core tube penetration, ultimately resulting in bacterial cell death (described below).

## Molecular structure of R-pyocins

Structurally, an R-pyocin particle is a contractile, syringe-like complex composed entirely of protein. It closely resembles a truncated bacteriophage tail, specifically being analogous to the tail of P2 phages. An R-pyocin consists of a hollow inner tube surrounded by a contractile sheath, a baseplate with attached tail fibres (also called receptor-binding fibres) at one end, and a sharp, iron-tipped tail spike at the end of the tube. The entire assembly is roughly 100–150 nm long (Fig. 1). The sheath is a spring-loaded structure held in a metastable, extended state by the baseplate; the tail fibres project from the baseplate and confer host specificity by binding to a particular LPS epitope on target bacteria. Notably, the R-pyocin sheath and tube are made of proteins orthologous to those in phage tails (e.g. 12 core proteins similar to phage T4 tail components), but there are no capsid or nucleic acid components included. The absence of DNA means R-pyocins are non-replicative, acting purely as one-time protein ‘nanomachines’ to kill cells. This also likely renders them impervious to anti-phage defences like Clustered Regularly Interspaced Short Palindromic Repeats (CRISPR) targeting of DNA, since no genetic material is injected.

The tail fibres of R-pyocins are homotrimeric proteins (~300–340 Å long) responsible for initial receptor recognition. The C-terminal region of the fibre determines receptor-binding specificity and thus defines the R-type subtype. Each tail fibre gene is associated with a small chaperone protein gene, which is required for proper folding of the fibre. This tight genomic coupling ensures efficient assembly of functional tail fibres. Overall, the architecture of R-pyocins is optimized for stability and lethality: they are robust, syringe-like complexes stable in the environment (no fragile phage head), loaded with the potential energy of the sheath and tipped with a membrane-piercing spike.

## Mechanism of action

R-pyocins exert their antibacterial effect through a highly specific membrane-puncturing mechanism similar to bacteriophage tail contraction, yet they cause cellular death via a distinctly different mechanism (Fig. 1). This mechanism operates via a rapid, single-hit mode, in which a single R-pyocin particle can lethally disrupt the target bacterial membrane, resulting in immediate cell depolarization and death. The killing process occurs through several sequential steps:

- **Receptor binding.** Initially, R-pyocin tail fibres specifically bind carbohydrate residues within the core oligosaccharide of the target bacterium’s LPS. Each R-pyocin subtype demonstrates affinity for distinct sugar moieties, such as inner-core glucose or rhamnose residues, providing remarkable specificity.
- **Sheath contraction and membrane penetration.** Activation of the baseplate triggers rapid contraction of the R-pyocin sheath, propelling a rigid internal tube through the outer membrane of the bacterial cell. The tube tip is hypothesized to possess a lysozyme-like domain (similar to T4-phage tails) derived from the dissociating tail-spike protein, enzymatically degrading the local peptidoglycan layer to facilitate deeper penetration into the periplasmic space.
- **Inner membrane (IM) disruption.** While complete puncture through the IM may not fully occur, close contact between the IM and the penetrating tube results in membrane deformation or invagination, effectively creating a transmembrane channel.
- **Depolarization and cell death.** Formation of the transmembrane channel leads to rapid ion efflux, triggering membrane potential collapse. The inner tube of the R-pyocin is highly negatively charged, promoting uncontrolled ionic movement and complete dissipation of the bacterial proton-motive force. The membrane potential swiftly falls from ~–90 to 0 mV, resulting in immediate cessation of ATP production and active transport mechanisms, thereby killing the bacterial cell. Notably, this killing mechanism likely functions independently of bacterial metabolic activity or growth state, enabling effective killing of dormant cells, thereby surpassing the limitations of traditional antibiotics and phage, which are often reliant on metabolic processes.

**Fig. 2.**
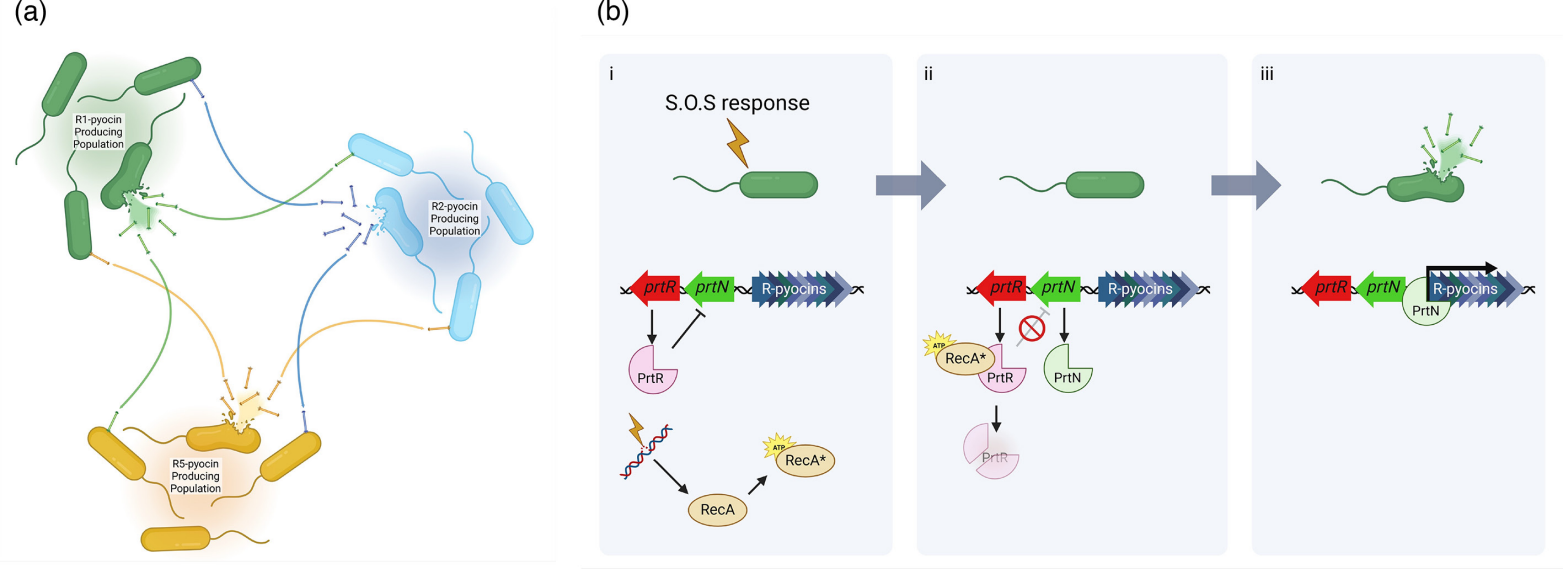
R-pyocin induction and R-pyocin-mediated strain interactions. (**a**) R-pyocins produced by *P. aeruginosa* strains are categorized into three subtypes, R1, R2 and R5. *P. aeruginosa* strains that produce an R-pyocin only produce R-pyocins of a single subtype, which are used to kill strains that produce different subtypes of R-pyocins. (**b**) Pyocin gene expression is induced via an SOS response. (**i**) DNA damage activates RecA. (ii) Activated RecA* cleaves PrtR, relieving the repression of the transcriptional regulator PrtB. (iii) PrtB upregulates transcription of the pyocin gene cluster, leading to cell lysis and the release of fully formed pyocins into the environment.

One notable characteristic of R-pyocins is the absence of dedicated immunity proteins typically found in other bacteriocins, such as S-pyocins or the colicins from *Escherichia coli*. It has been hypothesized that the producing strains inherently avoid self-destruction primarily due to modifications or differences in their LPS structure, which prevent R-pyocin binding and penetration. Upon release, R-pyocins indiscriminately kill neighbouring sensitive cells upon contact (Fig. 2). This physical killing modality significantly constrains possible resistance mechanisms; resistant strains typically must modify or obscure their LPS receptors or structurally reinforce their cell membranes. In chronic infections, *P. aeruginosa* isolates often evolve LPS core modifications or truncated O-antigen chains, perhaps as defensive strategies against R-pyocin killing and phage predation, albeit typically at the expense of reduced overall fitness or heightened vulnerability to immune clearance. The unique mechanistic attributes of R-pyocins (rapid lethality, single-hit efficacy and independence from bacterial physiological states) position them as compelling candidates for precision antibacterial therapy, particularly in the context of antibiotic-resistant bacterial infections.

## Genetic regulation of R-pyocin production

Genes encoding R-pyocins are organized in a distinct region of the chromosome, situated between the tryptophan biosynthesis genes *trpE* and *trpG*. These loci encompass structural genes necessary for the assembly of the baseplate, sheath, core tube, tail fibres and associated cell-lysis functions. Regulation of R-pyocin production involves a sophisticated system of regulatory genes, prominently including *prtR* and *prtN*, which orchestrate pyocin expression (Fig. 2). Under standard growth conditions, the *prtR* gene encodes a repressor protein that maintains suppression of the R-pyocin gene cluster. Upon encountering DNA damage or other stressors inducing the bacterial SOS response (e.g. UV radiation, certain antibiotics such as ciprofloxacin, or oxidative stress), the RecA protein becomes activated, leading to cleavage and inactivation of the PrtR repressor. The resultant release of repression enables PrtN, a positive regulatory factor, to initiate transcription of the entire R-pyocin gene cluster. This RecA-mediated regulatory mechanism aligns R-pyocin synthesis with conditions of cellular distress, strategically timing R-pyocin production to scenarios where competitive bacterial clearance may provide an adaptive advantage.

Recent studies indicate additional complexity in R-pyocin regulation, revealing alternative, RecA-independent pathways. Notably, *P. aeruginosa* mutants deficient in the site-specific recombinase XerC have shown elevated R-pyocin gene expression even in the absence of conventional SOS-triggering stimuli, though this induction still necessitates PrtN. These observations suggest that environmental or developmental signals, beyond DNA damage alone, can activate R-pyocin production through alternative regulatory routes.

Critically, the induction and subsequent release of R-pyocins are intrinsically linked to autolysis of the producing bacterial cell. Analogous to bacteriophage behaviour, the R-pyocin gene clusters include genes encoding lysis proteins (such as holins and endolysins), which facilitate cell rupture, releasing R-pyocins into the extracellular environment. This self-sacrificing mechanism, or altruistic lysis, ensures a high local concentration of R-pyocin particles, maximizing their antibacterial impact.

Intriguingly, certain *P. aeruginosa* strains harbour dual pyocin gene clusters (both R- and F-type pyocins), which share a unified regulatory framework mediated by PrtR and PrtN. This arrangement allows coordinated induction and concurrent release of multiple pyocin types, augmenting bacterial competitive strategies under stressful conditions.

## Therapeutic potential in antibiotic-resistant infections

R-pyocins have emerged as promising antimicrobial agents, particularly against challenging infections caused by antibiotic-resistant *P. aeruginosa*, such as those encountered in CF lung disease and healthcare-associated infections. Chronic *P. aeruginosa* infections often involve multidrug-resistant strains that form robust biofilms, significantly complicating treatment. R-pyocins offer a compelling therapeutic alternative or adjunct due to their unique bactericidal mechanisms and specificity. Key properties underscoring their therapeutic potential include:

- **High specificity.** R-pyocins target specific strains of *P. aeruginosa* via binding to distinct LPS receptor motifs, minimizing off-target effects on the commensal microbiota. This precise targeting mitigates the risk of dysbiosis typically associated with broad-spectrum antibiotic use.
- **Non-propagative nature.** Unlike bacteriophages, R-pyocins lack genetic material and therefore do not replicate, evolve or mediate gene transfer. They function strictly as proteinaceous antimicrobials, eliminating concerns related to phage propagation, such as genetic mobilization via horizontal gene transfer. Additionally, their proteinaceous nature likely renders them immune to bacterial anti-phage defence systems, such as CRISPR-Cas.
- **Rapid, irreversible killing.:** R-pyocins induce immediate bacterial death through rapid membrane depolarization, independent of the bacterial metabolic state or antibiotic-resistance profiles. This distinctive mechanism, involving membrane puncturing, significantly reduces the risk of cross-resistance with traditional antibiotics.
- **Engineering potential.** The modular nature of R-pyocins allows genetic manipulation of tail fibres, enabling retargeting towards different Gram-negative bacterial pathogens. This adaptability has been demonstrated by engineering chimeric R-pyocins incorporating bacteriophage tail fibre domains targeting *E. coli* O157:H7. Their intrinsic activity against non-*Pseudomonas* pathogens, such as certain *Neisseria* and *Haemophilus* species with similar LPS cores, further broadens their therapeutic scope.

Several studies have validated R-pyocin efficacy against *P. aeruginosa* strains isolated from patients with CF. Notably, variability in susceptibility among clonal variants from the same patient highlights the necessity for deploying mixtures or engineered R-pyocin variants targeting multiple LPS epitopes to comprehensively address chronic infections and mitigate potential resistance.

Preliminary *in vivo* and *ex vivo* evaluations reinforce their therapeutic feasibility. For instance, topical application of recombinant R-pyocins significantly reduced bacterial loads and disrupted biofilms in murine excision wound models without evident toxicity. For respiratory infections, particularly CF lung infections, inhalation-based therapies leveraging aerosolized or lyophilized formulations appear particularly promising. Encapsulation strategies, such as embedding R-pyocins in biocompatible microspheres or hydrogels, could enhance stability, protect against proteolytic degradation and facilitate sustained release.

Challenges for clinical translation include optimizing scalable production methods of pure R-pyocins, maintaining particle stability, minimizing immunogenicity upon systemic administration and formulating effective delivery mechanisms. Although R-pyocins demonstrate minimal immunogenicity in topical contexts, systemic applications necessitate careful evaluation of neutralizing antibody induction. Encouragingly, recombinant R-pyocins produced in heterologous hosts, such as *E. coli*, maintain full biological activity, simplifying large-scale manufacturing.

## Conclusion and outlook

R-type pyocins exemplify natural microbial competition strategies that can be repurposed into highly effective antibacterial agents. Detailed structural elucidation via cryo-electron microscopy has significantly advanced the understanding of their function, facilitating targeted bioengineering approaches. With the global rise of antibiotic resistance, the development of R-pyocin-based therapies, either alone or in combination with conventional antimicrobials, represents a compelling and innovative treatment strategy. Future clinical translation requires systematic validation of safety, efficacy, delivery methodologies, manufacturing reproducibility and regulatory compliance. Leveraging their specificity, potency and potential for genetic refinement positions R-pyocins as uniquely suited for tackling recalcitrant, chronic bacterial infections, particularly those involving antibiotic-resistant *P. aeruginosa*.

## Further reading

- Köhler T, Donner V, van Delden C. Lipopolysaccharide as shield and receptor for R-pyocin-mediated killing in *Pseudomonas aeruginosa*. *J Bacteriol* 2010;192:1921–1928. 10.1128/JB.01459-09
- Mei M, Thomas J, Diggle SP. Heterogenous susceptibilitySusceptibility to R-pyocinsPyocins in populationPopulations of *Pseudomonas aeruginosa* sourced from cystic fibrosis lSourced from Cystic Fibrosis Lungs. *mBio* 2021;12:e00458-21. 10.1128/mBio.00458-21
- Michel-Briand Y, Baysse C. The pyocins of *Pseudomonas aeruginosa*. *Biochimie* 2002;84:499–510. 10.1016/s0300-9084(02)01422-0
- Mei M, Estrada I, Diggle SP, Goldberg JB. R-pyocins as targeted antimicrobials against *Pseudomonas aeruginosa*. *NPJ Antimicrob Resist* 2025;3:17. 10.1038/s44259-025-00088-1
- Scholl D, Cooley M, Williams SR, Gebhart D, Martin D, *et al*. An engineered R-type pyocin is a highly specific and sensitive bactericidal agent for the food-borne pathogen *Escherichia coli* O157:H7. *Antimicrob Agents Chemother* 2009;53:3074–3080. 10.1128/AAC.01660-08
- Hu B, Margolin W, Molineux IJ, Liu J. Structural remodeling of bacteriophage T4 and host membranes during infection initiation. *Proc Natl Acad Sci USA* 2015;112:E4919–28. 10.1073/pnas.1501064112
- Govan JRW. Studies on the pyocinsPyocins of *Pseudomonas aeruginosa*: morphology and mode of action of contractile pyoMorphology and Mode of Action of Contractile Pyocins. *J Gen Microbiol* 1974;80:1–15. 10.1099/00221287-80-1-1
- Ge P, Scholl D, Prokhorov NS, Avaylon J, Shneider MM, *et al*. Action of a minimal contractile bactericidal nanomachine. *Nature* 2020;580:658–662. 10.1038/s41586-020-2186-z
- Alqahtani A, Kopel J, Hamood A. The *in vivo* and *in vitro* assessment of pyocins in treating *Pseudomonas aeruginosa* infectionsInfections. *Antibiotics* 2022;11:1366. 10.3390/antibiotics11101366
- Matsui H, Sano Y, Ishihara H, Shinomiya T. Regulation of pyocin genes in *Pseudomonas aeruginosa* by positive (prtN) and negative (prtR) regulatory genes. *J Bacteriol* 1993;175:1257–1263. 10.1128/jb.175.5.1257-1263.1993

## Abbreviations

CF: cystic fibrosis
IM: inner membrane

